# Acridin-10-ium 6-carb­oxy­pyridine-2-carboxyl­ate

**DOI:** 10.1107/S1600536811053578

**Published:** 2011-12-21

**Authors:** Kwang Ha

**Affiliations:** a School of Applied Chemical Engineering, The Research Institute of Catalysis, Chonnam National University, Gwangju 500-757, Republic of Korea

## Abstract

The title compound, C_13_H_10_N^+^·C_7_H_4_NO_4_^−^, consists of a protonated acridinium cation and a 6-carb­oxy­pyridine-2-carboxyl­ate monoanion. The carboxyl­ate group of the anion appears to be delocalized on the basis of the nearly equivalent C—O bond lengths. In the crystal, the anions are connected by strong O—H⋯O hydrogen bonds, forming chains along the *b* axis. The acridinium cations are linked to the anionic chains by strong N—H⋯O hydrogen bonds between the carboxyl­ate group of the anion and the N—H group of the cation. Along the *b* axis, successive chains stack in opposite directions. Weak inter­molecular C—H⋯O hydrogen bonds further stabilize the crystal structure.

## Related literature

For related crystal structures of acridinium compounds with carboxyl­ate, see: Shaameri *et al.* (2001 ▶); Derikvand *et al.* (2009 ▶); Attar Gharamaleki *et al.* (2010 ▶).
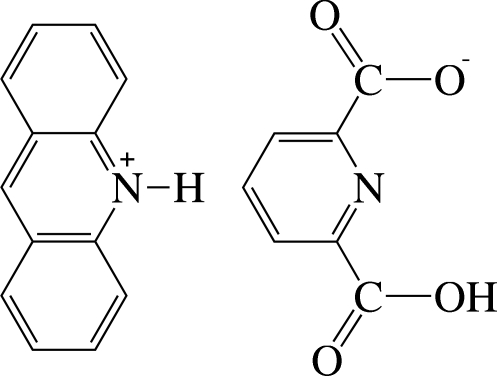


## Experimental

### 

#### Crystal data

C_13_H_10_N^+^·C_7_H_4_NO_4_^−^
*M* *_r_* = 346.33
Monoclinic, 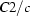

*a* = 16.6817 (8) Å
*b* = 8.2872 (4) Å
*c* = 23.7289 (12) Å
β = 105.582 (1)°
*V* = 3159.8 (3) Å^3^
*Z* = 8
Mo *K*α radiation
μ = 0.10 mm^−1^
*T* = 200 K
0.29 × 0.18 × 0.17 mm

#### Data collection

Bruker SMART 1000 CCD diffractometer
Absorption correction: multi-scan (*SADABS*; Bruker, 2000 ▶) *T*_min_ = 0.884, *T*_max_ = 1.000
11354 measured reflections
3894 independent reflections
2198 reflections with *I* > 2σ(*I*)
*R*_int_ = 0.046

#### Refinement

*R*[*F* ^2^ > 2σ(*F* ^2^)] = 0.051
*wR*(*F* ^2^) = 0.140
*S* = 1.06
3894 reflections
235 parameters
H-atom parameters constrained
Δρ_max_ = 0.31 e Å^−3^
Δρ_min_ = −0.27 e Å^−3^

### 

Data collection: *SMART* (Bruker, 2000 ▶); cell refinement: *SAINT* (Bruker, 2000 ▶); data reduction: *SAINT*; program(s) used to solve structure: *SHELXS97* (Sheldrick, 2008 ▶); program(s) used to refine structure: *SHELXL97* (Sheldrick, 2008 ▶); molecular graphics: *ORTEP-3* (Farrugia, 1997 ▶) and *PLATON* (Spek, 2009 ▶); software used to prepare material for publication: *SHELXL97*.

## Supplementary Material

Crystal structure: contains datablock(s) global, I. DOI: 10.1107/S1600536811053578/su2350sup1.cif

Structure factors: contains datablock(s) I. DOI: 10.1107/S1600536811053578/su2350Isup2.hkl

Supplementary material file. DOI: 10.1107/S1600536811053578/su2350Isup3.cml

Additional supplementary materials:  crystallographic information; 3D view; checkCIF report

## Figures and Tables

**Table:** 

O1—C19	1.314 (2)
O2—C19	1.206 (2)
O3—C20	1.255 (2)
O4—C20	1.246 (3)

**Table:** 

O2—C19—O1	124.6 (2)
O4—C20—O3	125.0 (2)

**Table 2:** Hydrogen-bond geometry (Å, °)

*D*—H⋯*A*	*D*—H	H⋯*A*	*D*⋯*A*	*D*—H⋯*A*
N1—H1*N*⋯O3^i^	0.92	1.70	2.602 (2)	165
O1—H1*O*⋯O4^ii^	0.84	1.73	2.535 (2)	160
C7—H7⋯O1^iii^	0.95	2.37	3.132 (2)	137
C10—H10⋯O3^iv^	0.95	2.49	3.435 (3)	171
C12—H12⋯O4^i^	0.95	2.56	3.466 (3)	160
C17—H17⋯O2^v^	0.95	2.44	3.387 (3)	171

Symmetry codes: (i) 
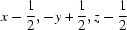
; (ii) 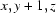; (iii) 
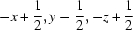
; (iv) 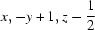; (v) 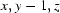.

**Fig. 1.**
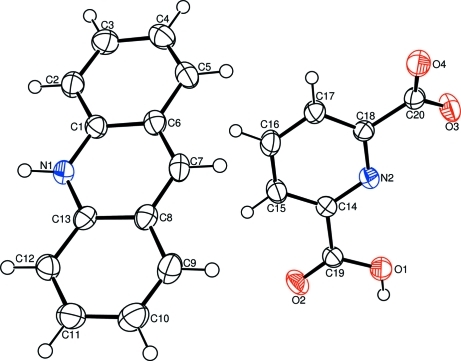
A structure detail of the title compound, with displacement ellipsoids drawn at the 50% probability level for non-H atoms.

**Fig. 2.**
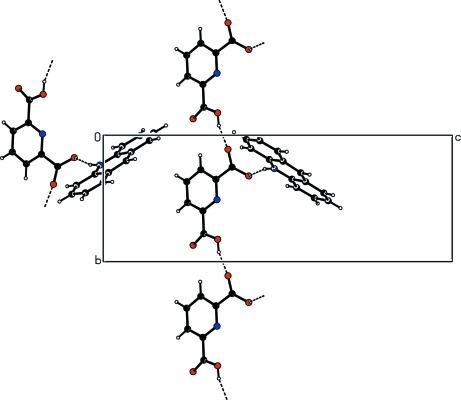
View of the unit-cell contents of the title compound. Hydrogen-bond interactions are drawn with dashed lines.

## supplementary crystallographic information

### Comment

Proton-transfer compounds of acridine and carboxylic acid, such as benzene-1,3-dicarboxylic acid (Shaameri *et al.*, 2001), benzene-1,3,5-tricarboxylic acid (Derikvand *et al.*, 2009) and pyrazine-2,3-dicarboxylic acid (Attar Gharamaleki *et al.*, 2010), have been investigated previously.

The title compound, C_13_H_10_N^+^.C_7_H_4_NO_4_^-^, consists of a protonated acridinium cation and a 6-carboxypyridine-2-carboxylate monoanion (Fig.1). The C—O bond lengths of the COOH group of the anion are somewhat different, but the C—O bond lengths of the carboxylate group are nearly equivalent (Table 1). On the basis of the C—O bond lengths, the carboxylate group appears to be delocalized. The O—C—O bond angles of the carboxylate and carboxy groups are almost equal (Table 1). In the crystal, the anions are connected by strong intermolecular O—H···O hydrogen bonds, forming one-dimensional chains along the *b* axis. The acridinium cations are linked to the anionic chains by strong intermolecular N—H···O hydrogen bonds between the carboxylate group of the anion and the NH group of the cation (Fig. 2 and Table 2). Along the *b* axis, successive chains stack in the opposite directions. Weak intermolecular C—H···O hydrogen bonds stabilize also the crystal structure (Table 2). Numerous intermolecular π-π interactions are also present between adjacent six-membered rings, the shortest centroid-centroid distance being 3.734 (1) Å.

### Experimental

To a solution of acridine (0.3584 g, 2.00 mmol) in EtOH (20 ml) was added pyridine-2,6-dicarboxylic acid (0.1671 g, 1.00 mmol) and refluxed for 3 h. The formed precipitate was separated by filtration, washed with ether and dried at 50 °C, to give a yellow powder (0.2281 g). Crystals suitable for X-ray diffraction analysis were obtained by slow evaporation from a water solution.

### Refinement

Carbon-bound H atoms were positioned geometrically and allowed to ride on their respective parent atoms [C—H = 0.95 Å and *U*_iso_(H) = 1.2*U*_eq_(C)]. Nitrogen- and oxygen-bound H atoms were located from Fourier difference maps then allowed to ride on their parent atoms in the final cycles of refinement with N—H = 0.92 Å, O—H = 0.84 Å and *U*_iso_(H) = 1.5 *U*_eq_(N, O). The highest peak (0.31 e Å^-3^) and the deepest hole (-0.27 e Å^-3^) in the difference Fourier map are located 0.68 Å and 1.11 Å from the atoms C6 and C15, respectively.

### Crystal data

**Table:**

C_13_H_10_N^+^·C_7_H_4_NO_4_^−^	*F*(000) = 1440
*M**_r_* = 346.33	*D*_x_ = 1.456 Mg m^−^^3^
Monoclinic, *C*2/*c*	Mo *K*α radiation, λ = 0.71073 Å
Hall symbol: -C 2yc	Cell parameters from 3018 reflections
*a* = 16.6817 (8) Å	θ = 2.5–27.8°
*b* = 8.2872 (4) Å	µ = 0.10 mm^−^^1^
*c* = 23.7289 (12) Å	*T* = 200 K
β = 105.582 (1)°	Block, yellow
*V* = 3159.8 (3) Å^3^	0.29 × 0.18 × 0.17 mm
*Z* = 8	

### Data collection

**Table:**

Bruker SMART 1000 CCD diffractometer	3894 independent reflections
Radiation source: fine-focus sealed tube	2198 reflections with *I* > 2σ(*I*)
graphite	*R*_int_ = 0.046
φ and ω scans	θ_max_ = 28.3°, θ_min_ = 2.5°
Absorption correction: multi-scan (*SADABS*; Bruker, 2000)	*h* = −22→19
*T*_min_ = 0.884, *T*_max_ = 1.000	*k* = −10→11
11354 measured reflections	*l* = −29→31

### Refinement

**Table:**

Refinement on *F*^2^	Primary atom site location: structure-invariant direct methods
Least-squares matrix: full	Secondary atom site location: difference Fourier map
*R*[*F*^2^ > 2σ(*F*^2^)] = 0.051	Hydrogen site location: inferred from neighbouring sites
*wR*(*F*^2^) = 0.140	H-atom parameters constrained
*S* = 1.06	*w* = 1/[σ^2^(*F*_o_^2^) + (0.0484*P*)^2^ + 0.8669*P*] where *P* = (*F*_o_^2^ + 2*F*_c_^2^)/3
3894 reflections	(Δ/σ)_max_ < 0.001
235 parameters	Δρ_max_ = 0.31 e Å^−^^3^
0 restraints	Δρ_min_ = −0.27 e Å^−^^3^

### Special details

**Table:**

Geometry. All e.s.d.'s (except the e.s.d. in the dihedral angle between two l.s. planes) are estimated using the full covariance matrix. The cell e.s.d.'s are taken into account individually in the estimation of e.s.d.'s in distances, angles and torsion angles; correlations between e.s.d.'s in cell parameters are only used when they are defined by crystal symmetry. An approximate (isotropic) treatment of cell e.s.d.'s is used for estimating e.s.d.'s involving l.s. planes.
Refinement. Refinement of *F*^2^ against ALL reflections. The weighted *R*-factor *wR* and goodness of fit *S* are based on *F*^2^, conventional *R*-factors *R* are based on *F*, with *F* set to zero for negative *F*^2^. The threshold expression of *F*^2^ > σ(*F*^2^) is used only for calculating *R*-factors(gt) *etc*. and is not relevant to the choice of reflections for refinement. *R*-factors based on *F*^2^ are statistically about twice as large as those based on *F*, and *R*- factors based on ALL data will be even larger.

### Fractional atomic coordinates and isotropic or equivalent isotropic displacement parameters (Å^2^)

**Table:**

	*x*	*y*	*z*	*U*_iso_*/*U*_eq_	
N1	−0.04881 (10)	0.2361 (2)	−0.00985 (7)	0.0305 (4)	
H1N	−0.1001	0.2338	−0.0367	0.046*	
C1	−0.04063 (12)	0.1529 (2)	0.04054 (9)	0.0300 (5)	
C2	−0.11101 (13)	0.0861 (3)	0.05417 (10)	0.0369 (5)	
H2	−0.1648	0.0986	0.0280	0.044*	
C3	−0.10107 (14)	0.0039 (3)	0.10497 (10)	0.0410 (6)	
H3	−0.1486	−0.0397	0.1143	0.049*	
C4	−0.02137 (14)	−0.0185 (3)	0.14456 (10)	0.0408 (6)	
H4	−0.0161	−0.0766	0.1799	0.049*	
C5	0.04717 (14)	0.0427 (3)	0.13213 (9)	0.0366 (5)	
H5	0.1005	0.0258	0.1584	0.044*	
C6	0.03965 (13)	0.1322 (3)	0.07986 (9)	0.0308 (5)	
C7	0.10755 (13)	0.2029 (3)	0.06581 (9)	0.0338 (5)	
H7	0.1617	0.1884	0.0912	0.041*	
C8	0.09735 (12)	0.2943 (3)	0.01523 (9)	0.0317 (5)	
C9	0.16393 (14)	0.3735 (3)	−0.00016 (10)	0.0413 (6)	
H9	0.2183	0.3690	0.0257	0.050*	
C10	0.15110 (15)	0.4556 (3)	−0.05128 (11)	0.0446 (6)	
H10	0.1965	0.5060	−0.0613	0.054*	
C11	0.07003 (14)	0.4662 (3)	−0.08980 (10)	0.0408 (6)	
H11	0.0618	0.5243	−0.1254	0.049*	
C12	0.00364 (14)	0.3949 (3)	−0.07683 (9)	0.0344 (5)	
H12	−0.0504	0.4037	−0.1029	0.041*	
C13	0.01634 (12)	0.3078 (2)	−0.02393 (9)	0.0294 (5)	
O1	0.24957 (9)	0.82325 (18)	0.32785 (7)	0.0433 (4)	
H1O	0.2533	0.9241	0.3311	0.065*	
O2	0.13288 (10)	0.87006 (19)	0.25747 (7)	0.0475 (5)	
O3	0.30738 (9)	0.32253 (19)	0.41697 (7)	0.0431 (4)	
O4	0.29134 (9)	0.11156 (19)	0.35743 (7)	0.0462 (5)	
N2	0.22360 (10)	0.5100 (2)	0.32603 (7)	0.0295 (4)	
C14	0.17072 (12)	0.5985 (3)	0.28554 (9)	0.0295 (5)	
C15	0.10598 (13)	0.5332 (3)	0.24202 (10)	0.0369 (5)	
H15	0.0686	0.6007	0.2148	0.044*	
C16	0.09746 (14)	0.3678 (3)	0.23931 (10)	0.0407 (6)	
H16	0.0540	0.3192	0.2099	0.049*	
C17	0.15273 (13)	0.2735 (3)	0.27968 (9)	0.0346 (5)	
H17	0.1489	0.1591	0.2779	0.041*	
C18	0.21413 (12)	0.3490 (2)	0.32294 (9)	0.0284 (5)	
C19	0.18181 (13)	0.7786 (3)	0.28826 (9)	0.0315 (5)	
C20	0.27569 (12)	0.2525 (3)	0.36928 (10)	0.0317 (5)	

### Atomic displacement parameters (Å^2^)

**Table:**

	*U*^11^	*U*^22^	*U*^33^	*U*^12^	*U*^13^	*U*^23^
N1	0.0284 (9)	0.0307 (10)	0.0287 (10)	−0.0019 (8)	0.0014 (8)	−0.0006 (8)
C1	0.0333 (11)	0.0281 (12)	0.0270 (11)	0.0005 (9)	0.0054 (9)	−0.0018 (9)
C2	0.0321 (12)	0.0428 (14)	0.0339 (13)	−0.0032 (10)	0.0056 (10)	0.0001 (11)
C3	0.0422 (13)	0.0437 (15)	0.0396 (14)	−0.0047 (11)	0.0153 (11)	0.0029 (11)
C4	0.0491 (14)	0.0400 (14)	0.0331 (13)	0.0023 (11)	0.0106 (11)	0.0059 (10)
C5	0.0410 (13)	0.0360 (13)	0.0290 (12)	0.0066 (10)	0.0030 (10)	0.0012 (10)
C6	0.0328 (11)	0.0285 (12)	0.0289 (12)	0.0023 (9)	0.0048 (9)	−0.0046 (9)
C7	0.0286 (11)	0.0379 (13)	0.0300 (12)	0.0006 (10)	−0.0005 (9)	−0.0045 (10)
C8	0.0315 (11)	0.0307 (12)	0.0313 (12)	−0.0033 (9)	0.0055 (9)	−0.0052 (10)
C9	0.0309 (12)	0.0485 (15)	0.0416 (14)	−0.0053 (11)	0.0045 (10)	−0.0037 (12)
C10	0.0423 (14)	0.0479 (16)	0.0468 (15)	−0.0110 (12)	0.0173 (11)	−0.0029 (12)
C11	0.0490 (14)	0.0371 (14)	0.0369 (14)	−0.0043 (11)	0.0124 (11)	0.0029 (11)
C12	0.0377 (12)	0.0328 (13)	0.0303 (12)	−0.0004 (10)	0.0047 (10)	−0.0012 (10)
C13	0.0294 (11)	0.0262 (12)	0.0319 (12)	−0.0030 (9)	0.0072 (9)	−0.0048 (9)
O1	0.0412 (9)	0.0265 (9)	0.0531 (11)	0.0000 (7)	−0.0029 (8)	0.0003 (7)
O2	0.0533 (10)	0.0325 (9)	0.0460 (10)	0.0081 (8)	−0.0049 (8)	0.0074 (8)
O3	0.0454 (9)	0.0406 (10)	0.0344 (9)	0.0111 (8)	−0.0047 (7)	−0.0041 (8)
O4	0.0396 (9)	0.0277 (9)	0.0617 (12)	0.0043 (7)	−0.0029 (8)	−0.0070 (8)
N2	0.0277 (9)	0.0319 (10)	0.0283 (10)	0.0035 (8)	0.0063 (7)	0.0020 (8)
C14	0.0302 (11)	0.0292 (12)	0.0292 (12)	0.0011 (9)	0.0082 (9)	0.0007 (9)
C15	0.0375 (12)	0.0381 (14)	0.0305 (12)	0.0037 (10)	0.0010 (10)	0.0012 (10)
C16	0.0375 (12)	0.0389 (14)	0.0375 (14)	−0.0019 (11)	−0.0040 (10)	−0.0047 (11)
C17	0.0349 (12)	0.0292 (12)	0.0354 (13)	−0.0003 (10)	0.0021 (10)	−0.0034 (10)
C18	0.0276 (10)	0.0262 (12)	0.0313 (12)	0.0005 (9)	0.0077 (9)	−0.0008 (9)
C19	0.0330 (11)	0.0324 (12)	0.0285 (12)	0.0009 (10)	0.0071 (9)	0.0023 (10)
C20	0.0270 (11)	0.0295 (13)	0.0371 (13)	0.0002 (9)	0.0057 (10)	−0.0005 (10)

### Geometric parameters (Å, °)

**Table:**

N1—C1	1.355 (3)	C10—H10	0.9500
N1—C13	1.357 (3)	C11—C12	1.361 (3)
N1—H1N	0.9200	C11—H11	0.9500
C1—C2	1.412 (3)	C12—C13	1.414 (3)
C1—C6	1.422 (3)	C12—H12	0.9500
C2—C3	1.355 (3)	O1—C19	1.314 (2)
C2—H2	0.9500	O1—H1O	0.8400
C3—C4	1.419 (3)	O2—C19	1.206 (2)
C3—H3	0.9500	O3—C20	1.255 (2)
C4—C5	1.354 (3)	O4—C20	1.246 (3)
C4—H4	0.9500	N2—C14	1.335 (3)
C5—C6	1.421 (3)	N2—C18	1.344 (3)
C5—H5	0.9500	C14—C15	1.388 (3)
C6—C7	1.393 (3)	C14—C19	1.503 (3)
C7—C8	1.390 (3)	C15—C16	1.378 (3)
C7—H7	0.9500	C15—H15	0.9500
C8—C9	1.420 (3)	C16—C17	1.380 (3)
C8—C13	1.424 (3)	C16—H16	0.9500
C9—C10	1.357 (3)	C17—C18	1.389 (3)
C9—H9	0.9500	C17—H17	0.9500
C10—C11	1.417 (3)	C18—C20	1.515 (3)
			
C1—N1—C13	122.86 (17)	C12—C11—C10	121.4 (2)
C1—N1—H1N	117.1	C12—C11—H11	119.3
C13—N1—H1N	119.7	C10—C11—H11	119.3
N1—C1—C2	120.59 (18)	C11—C12—C13	119.0 (2)
N1—C1—C6	119.62 (19)	C11—C12—H12	120.5
C2—C1—C6	119.8 (2)	C13—C12—H12	120.5
C3—C2—C1	119.3 (2)	N1—C13—C12	120.22 (18)
C3—C2—H2	120.3	N1—C13—C8	119.18 (19)
C1—C2—H2	120.3	C12—C13—C8	120.60 (19)
C2—C3—C4	121.6 (2)	C19—O1—H1O	112.0
C2—C3—H3	119.2	C14—N2—C18	117.44 (18)
C4—C3—H3	119.2	N2—C14—C15	123.6 (2)
C5—C4—C3	120.2 (2)	N2—C14—C19	117.66 (19)
C5—C4—H4	119.9	C15—C14—C19	118.77 (19)
C3—C4—H4	119.9	C16—C15—C14	118.2 (2)
C4—C5—C6	120.3 (2)	C16—C15—H15	120.9
C4—C5—H5	119.9	C14—C15—H15	120.9
C6—C5—H5	119.9	C15—C16—C17	119.3 (2)
C7—C6—C5	122.78 (19)	C15—C16—H16	120.3
C7—C6—C1	118.4 (2)	C17—C16—H16	120.3
C5—C6—C1	118.8 (2)	C16—C17—C18	118.7 (2)
C8—C7—C6	121.12 (19)	C16—C17—H17	120.6
C8—C7—H7	119.4	C18—C17—H17	120.6
C6—C7—H7	119.4	N2—C18—C17	122.70 (19)
C7—C8—C9	123.4 (2)	N2—C18—C20	115.95 (18)
C7—C8—C13	118.73 (19)	C17—C18—C20	121.34 (19)
C9—C8—C13	117.8 (2)	O2—C19—O1	124.6 (2)
C10—C9—C8	121.0 (2)	O2—C19—C14	122.9 (2)
C10—C9—H9	119.5	O1—C19—C14	112.50 (18)
C8—C9—H9	119.5	O4—C20—O3	125.0 (2)
C9—C10—C11	120.1 (2)	O4—C20—C18	118.25 (19)
C9—C10—H10	119.9	O3—C20—C18	116.73 (19)
C11—C10—H10	119.9		
			
C13—N1—C1—C2	177.4 (2)	C11—C12—C13—N1	179.8 (2)
C13—N1—C1—C6	−2.9 (3)	C11—C12—C13—C8	−0.1 (3)
N1—C1—C2—C3	−179.8 (2)	C7—C8—C13—N1	2.3 (3)
C6—C1—C2—C3	0.5 (3)	C9—C8—C13—N1	−178.68 (19)
C1—C2—C3—C4	−0.8 (4)	C7—C8—C13—C12	−177.8 (2)
C2—C3—C4—C5	0.0 (4)	C9—C8—C13—C12	1.2 (3)
C3—C4—C5—C6	1.1 (4)	C18—N2—C14—C15	1.8 (3)
C4—C5—C6—C7	177.6 (2)	C18—N2—C14—C19	−179.12 (18)
C4—C5—C6—C1	−1.4 (3)	N2—C14—C15—C16	−2.2 (3)
N1—C1—C6—C7	1.8 (3)	C19—C14—C15—C16	178.8 (2)
C2—C1—C6—C7	−178.5 (2)	C14—C15—C16—C17	0.3 (4)
N1—C1—C6—C5	−179.16 (19)	C15—C16—C17—C18	1.7 (3)
C2—C1—C6—C5	0.6 (3)	C14—N2—C18—C17	0.4 (3)
C5—C6—C7—C8	−177.7 (2)	C14—N2—C18—C20	179.50 (18)
C1—C6—C7—C8	1.2 (3)	C16—C17—C18—N2	−2.2 (3)
C6—C7—C8—C9	177.8 (2)	C16—C17—C18—C20	178.8 (2)
C6—C7—C8—C13	−3.2 (3)	N2—C14—C19—O2	−172.6 (2)
C7—C8—C9—C10	177.2 (2)	C15—C14—C19—O2	6.5 (3)
C13—C8—C9—C10	−1.8 (3)	N2—C14—C19—O1	6.8 (3)
C8—C9—C10—C11	1.3 (4)	C15—C14—C19—O1	−174.07 (19)
C9—C10—C11—C12	−0.2 (4)	N2—C18—C20—O4	−153.4 (2)
C10—C11—C12—C13	−0.4 (3)	C17—C18—C20—O4	25.7 (3)
C1—N1—C13—C12	−179.1 (2)	N2—C18—C20—O3	26.3 (3)
C1—N1—C13—C8	0.8 (3)	C17—C18—C20—O3	−154.6 (2)

### Hydrogen-bond geometry (Å, °)

**Table:**

*D*—H···*A*	*D*—H	H···*A*	*D*···*A*	*D*—H···*A*
N1—H1N···O3^i^	0.92	1.70	2.602 (2)	165.
O1—H1O···O4^ii^	0.84	1.73	2.535 (2)	160.
C7—H7···O1^iii^	0.95	2.37	3.132 (2)	137.
C10—H10···O3^iv^	0.95	2.49	3.435 (3)	171.
C12—H12···O4^i^	0.95	2.56	3.466 (3)	160.
C17—H17···O2^v^	0.95	2.44	3.387 (3)	171.

Symmetry codes: (i) *x*−1/2, −*y*+1/2, *z*−1/2; (ii) *x*, *y*+1, *z*; (iii) −*x*+1/2, *y*−1/2, −*z*+1/2; (iv) *x*, −*y*+1, *z*−1/2; (v) *x*, *y*−1, *z*.
